# The effectiveness and cost-effectiveness of motion style acupuncture treatment (MSAT) for acute neck pain: A multi-center randomized controlled trial

**DOI:** 10.1097/MD.0000000000022871

**Published:** 2020-10-30

**Authors:** Doori Kim, Yoon Jae Lee, Kyoung Sun Park, Suna Kim, Ji-Yeon Seo, Hyun Woo Cho, In-Hyuk Ha

**Affiliations:** a Jaseng Spine and Joint Research Institute, Jaseng Medical Foundation; b Jaseng Hospital of Korean Medicine, Gangnam-gu, Seoul; c Daejeon Jaseng Hospital of Korean Medicine, Seo-gu, Daejeon; d Bucheon Jaseng Hospital of Korean Medicine, Bucheon, Gyeonggi-do; e Haeundae Jaseng Hospital of Korean Medicine, Haeundae-gu, Busan, Republic of Korea.

**Keywords:** acupuncture, acute neck pain, motion style acupuncture treatment, protocol, randomized controlled trial

## Abstract

**Background:**

Neck pain is a common complaint in the general population. Despite the consistent ongoing pain and the resulting economic burden on affected individuals, there have only been a few studies investigating the treatment of acute neck pain. This study aims to evaluate the effectiveness, safety, and cost-effectiveness of the motion style acupuncture treatment (MSAT) and acupuncture treatment for acute neck pain.

**Methods:**

This 2-armed, parallel, multi-centered randomized controlled trial will be conducted at 4 community-based hospitals in Korea. A total of 128 subjects will be randomly assigned, at a 1:1 ratio, to the MSAT and the acupuncture treatment groups. Treatment will be administered 2 to 3 times a week for 2 weeks. The primary outcome will be the visual analog scale of neck pain on movement. The secondary outcomes will be the numeric rating scale of the neck, neck disability index, Northwick Park questionnaire, patient global impression of change, range of motion of the neck, 5-level EuroQol-5 dimension, 12-item Short-Form Health Survey, and EuroQol visual analogue scale. This protocol has been registered at the Clinicaltrials.gov (NCT04539184).

**Discussion:**

To our knowledge, this study is the first well-designed multi-centered randomized controlled trial to evaluate the effectiveness, safety, and cost-effectiveness of MSAT on acute neck pain. The results of this study will be useful for clinicians in primary medical institutions that frequently treat acute neck pain patients and for policymakers working with national health insurance.

## 1 Introduction

Neck pain is one of the common musculoskeletal disorders in the general population. Although the prevalence of neck pain varies by studies conducted and the time period, the 1-year prevalence rate is reportedly 25.8%^[1]^ and the prevalence in an adult's lifetime is 50%.^[2]^ Neck pain can develop into considerably severe pain, disability, and cause a financial burden.^[3]^ Years lived with disability from neck pain per 100,000 population was reportedly 352 from 1990 to 2017,^[4]^ and the neck pain was the fourth main cause of years lived with disability according to the 2016 Global Burden of Disease.^[5]^

Among the different types of neck pains, acute neck pain is considered a self-limiting disorder and adults can experience pain during typical activities (e.g., lifting, twisting, and stretching). Acute neck pain is caused by loads applied internally to the tissues or external loads such as a fall and direct impact to the head or neck. However, little is known about the prevalence and treatment of acute neck pain, especially those not caused by whiplash. Vernon et al^[6]^ reported that the point prevalence of acute neck pain to be around 10%. There is currently no consensus for the definition of acute neck pain but is often defined as pain that does not last for more than 4 weeks.^[7,8]^ There is no standardized guideline for the treatment of acute neck pain but in a study of patients that visited general practitioners for acute neck pain in the Netherlands, 42% of patients were prescribed non-steroidal anti-inflammatory drugs and muscle relaxation.^[9]^ In addition, other studies have reported on the effectiveness of laser therapy^[10]^ and cervical spine thrust manipulation^[11]^ on acute neck pain.

The motion-style acupuncture technique (MSAT), which combines traditional acupuncture and movement therapy, is a treatment that induces passive and active movements of the patient with the needles inserted, after a traditional acupuncture treatment. All movements are made under the observation and guidance of a physician. MSAT has been increasingly used to relieve musculoskeletal pain in Korean clinical practice and recently has gained attraction in China as a method to enhance the effect of traditional acupuncture treatment.^[12–14]^ However, the supporting evidence and data for the effectiveness and safety of MSAT are insufficient as only a few related studies have been conducted.

Recently, the combination of MSAT and integrative Korean medicine treatment was reported to be effective for rapid pain reduction and range of motion (ROM) improvement in patients with pain in the cervical spine region due to acute whiplash injury.^[15]^ However, the study was only conducted for patients with a whiplash injury due to a traffic accident and the effectiveness of MSAT alone could not be examined since it was in a combination with the integrative Korean medicine treatment. Therefore, this 2-parallel, multi-centered randomized trial has been designed to evaluate the effectiveness, safety, and cost-effectiveness of MSAT and acupuncture treatment for acute neck pain that has not been caused by whiplash injury.

## 2 Methods

### 2.1 Study design and setting (Fig. 1 and Table 1)

**Figure 1:**
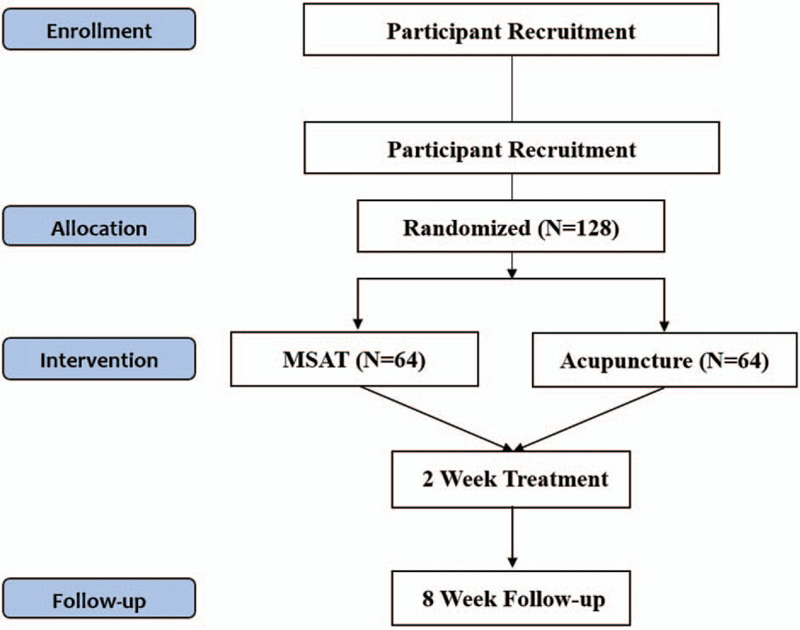
Study flow diagram.

This 2-armed, parallel, multi-centered randomized controlled trial (RCT) will be conducted at four community-based hospitals in Korea; Jaseng Hospital of Korean Medicine, Daejeon Jaseng Hospital of Korean Medicine, Bucheon Jaseng Hospital of Korean Medicine, and Haeundae Jaseng Hospital of Korean Medicine. A total of 128 patients, 64 for the MSAT and 64 for the acupuncture treatment groups, will be recruited from the 4 hospitals. Treatment will be performed 2 to 3 times a week for 2 weeks, followed by a follow-up for 8 weeks after randomization (Fig. 1).

**Table 1:**
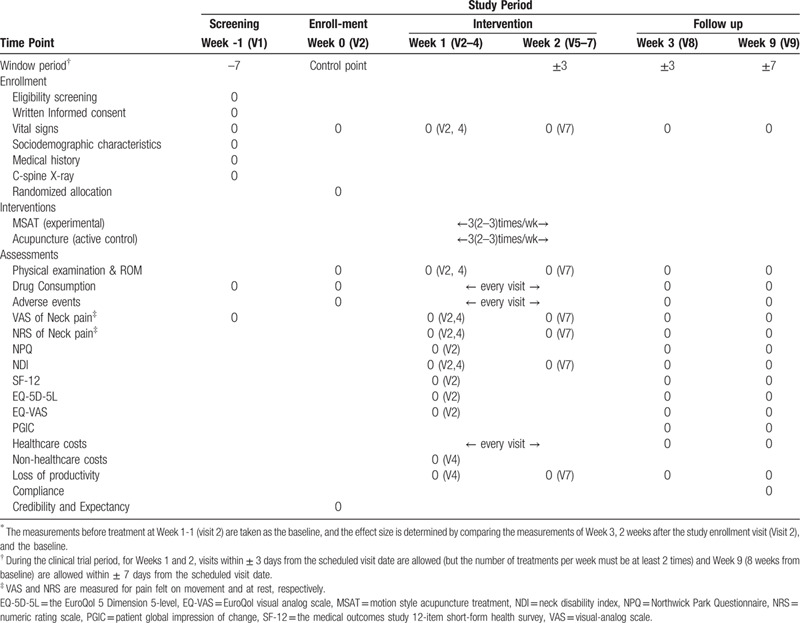
The timeline of participants^∗^.

The study protocol was approved by the institutional review board (IRB), at each of the institutions, before initiating the participant recruitment process (JASENG 2020-07-014, JASENG 2020-07-015, JASENG 2020-07-016, and JASENG 2020-07-017). The protocol has been registered at the Clinicaltrials.gov (NCT04539184). Information regarding the healthcare institutions and investigators can be found on the clinical trial registration website.

### 2.2 Participants

#### 2.2.1 Inclusion criteria

1) Neck pain onset or worsening within 1 month
2) Visual Analog Scale (VAS) of neck pain on movement or at rest of 5 or higher
3) Age between 19 and 70 years old
4) Participants that provided informed consent

#### 2.2.2 Exclusion criteria

1) Participants diagnosed with a serious disease that can cause pain (e.g., Migration of cancer reaching to spine, fracture of the spine, and dislocation of the spine)
2) Progressive neurologic deficits or severe neurologic deficits
3) Participants diagnosed with a soft tissue disease that can cause pain (Cancer, fibromyalgia, rheumatoid arthritis, or goat)
4) Presence of chronic diseases (e.g., Cardiovascular disease, kidney disease, dementia, diabetic neuropathy, or epilepsy)
5) Participants on steroids, immunosuppressants, or psychotropic medication
6) Participants who are inadequate or unsafe for acupuncture (e.g., Hemorrhagic disease, severe diabetes, or taking the anticoagulant drug)
7) Participants that took NSAIDs or underwent acupuncture treatment within the last 3 days
8) Participants that had undergone cervical surgery within the last 3 months
9) Participants that had a traffic accident within a month
10) Pregnant women or women who planned to conceive
11) Participants that had participated in another clinical trial within 1 month or planned to participle in other trials within 6 months of the enrollment
12) Participants that could not provide informed consent
13) Participants that found it difficult to participate in the trial according to the investigator's decision

The patient may be dropped in clinical trials in the case of an unexpected risk or serious adverse events are occurred. The PI would make the final decision if to drop the patient.

### 2.3 Interventions

All treatments, including the MSAT and acupuncture treatment, will be administered by Korean medicine doctors who have more than 3 years of clinical experience and received standardized training on both the Korean medicine treatment and MSAT. All treatments will be carried out for a total of 2 weeks.

#### 2.3.1 Experimental group: MSAT

The protocol for trapezius MSAT is as follows; the patient sits on a chair or the floor, straightens from low back up to the neck, pulls their chin slightly toward the body, and directs their gaze towards the front. In this position, the physician rotates the neck alternately from left to right with 3 disposable needles (30 mm ∗ 0.25 mm; Dong-bang Acupuncture, Korea) inserted into the upper trapezius on both sides of the subject at a depth of about 5 to 10 mm. The neck movement range shall only be up to the ROM and the physician examines whether there is a difference from the normal ROM and presence of other abnormalities in the movement. When the side with the smaller left-to-right ROM is set as the affected side, the left-to-right turning motion is performed regarding the ROM on the affected side. At this time, with the midline of the body as the reference, the patient breathes in when moving closer to the midline and breathes out when moving away from the midline. The above movements are repeated 8 to 10 times and the physician examines whether the movement of the patient's head and neck area is normal. In case of a problem with the movement, the physician guides the patient by placing 1 hand on the posterior cervical region and the other hand on the face, thereby correcting the movement. After completing the repetitive movements, the maximum ROM is checked again and if it does not achieve normal ROM, the left-to-right turning motion is repeated. The procedure continues for a specified treatment time (within 15 minutes) and at the discretion of the physician, the treatment process could either be continued or undergo only the movement therapy or the acupuncture treatment without movement (additional acupoints if necessary) after the treatment process.

A total of 6 treatments will be performed 3 times a week for 2 weeks; however, depending on the patient's condition or circumstances, a total of 4 to 6 treatments for trapezius MSAT may be performed during 2 weeks.

#### 2.3.2 Active control group: acupuncture

The protocol for trapezius acupuncture is as follows; Acupuncture points are selected from primary meridians (SI15, TE15, and LI16 bilateral) and extraordinary meridians (GB20, BL10, SI14 bilateral, and GV14 unilateral) on the bilateral upper and middle trapezius according to the physician's discretion. Acupuncture is performed with needles inserted into 6 to 12 selected points. Disposable needles (30 mm ∗ 0.25 mm; Dong-bang Acupuncture, Korea) are used for the insertion, and depending on the characteristics of the acupuncture point, after the perpendicular or oblique insertion, a twirling method is performed for the patient to feel the acupuncture sensation.

A total of 6 treatments will be performed 3 times a week for 2 weeks; however, depending on the patient's condition or circumstances, a total of 4 to 6 treatments may be performed when conducting the treatment twice a week. Duration for the needle insertion will be approximately 15 minutes.

#### 2.3.3 Relevent concomitant care

All treatments (drugs, surgery, nerve block, acupuncture, physical therapy, etc.) for relieve neck pain are prohibited until the 3^rd^ week (visit 9), the primary endpoint in both MSAT and control groups. There are no treatment restrictions during the f/u period after the primary endpoint. However, during the study period, acetaminophen (maximum 4 g per day) will be allowed to all subjects and the use of the drug will be recorded in a self-report method.

### 2.4 Outcome

#### 2.4.1 Primary outcome: visual analogue scale on movement (VAS on movement)

The primary outcome of this study will be the difference between the baseline (Week 1) and the primary endpoint, week 3 (visit 8) for the neck pain felt during movement. The VAS is an assessment tool on which the patient indicates the level of pain along a continuous line of 100 mm in length, with one end indicating no pain and the other indicating the worst pain possible.^[16,17]^

The VAS of neck pain felt on movement will be measured 7 times in total; at the screening, baseline, the last visit of each week during the intervention period (visits 4 and 7), week 3 (visit 8), and week 8 (visit 9).

#### 2.4.2 Secondary outcomes

##### 2.4.2.1 Visual analogue scale at rest (VAS at rest)

The VAS of neck pain felt at rest will be measured 6 times in total; at the baseline, the last visit of each week during the intervention period (visits 4 and 7), week 3 (visit 8), and week 8 (visit 9).

##### 2.4.2.2 Numeric rating scale (NRS)

NRS is a numeric pain scale for an objective assessment of subjective pain felt by patients.^[16,18]^ The patient selects the appropriate point, with no pain as 0 and the worst pain possible as 10. The level of pain felt by the patient on movement and at rest will be assessed.

NRS will be measured 6 times in total; at the baseline, the last visit of each week during the intervention period (visits 4 and 7), week 3 (visit 8), and week 8 (visit 9).

##### 2.4.2.3 Vernon-Mior neck disability index (NDI)

NDI is an assessment tool for disabilities caused by neck pain while performing daily activities and consists of 10 items. Scores from 0 to 5 can be selected for each item, allowing for a total score of 50 points. The higher the score, the greater the disability in daily living.^[19,20]^

NDI will be measured 6 times in total; at the baseline, the last visit of each week during the intervention period (visits 4 and 7), week 3 (visit 8), and week 8 (visit 9).

##### 2.4.2.4 Northwick park neck pain questionnaire (NPQ)

The NPQ is a questionnaire that consists of 9-question items on daily life activities affected by the neck pain (intensity, sleep, numbness, duration, carrying things, reading/watching TV, working, social activities, and driving). Each question consists of 5 levels with 0 to 4 points, and a higher score indicates more severe consequent patient disabilities.^[21]^ In this study, the Korean version of the NPQ, translated and validated by Lee at al, will be used.^[22]^

NPQ will be measured 3 times in total; at the baseline, week 3 (visit 8), and week 8 (visit 9).

##### 2.4.2.5 Patient global impression of change (PGIC)

PGIC is a scale that assesses the patient's perception of improvement in 7 stages, and the subject responds to the improvement in functional limitations after treatment with a 7-point Likert scale. (1 = very much improved, 2 = much improved, 3 = minimally improved, 4 = no change, 5 = minimally worse, 6 = much worse, and 7 = very much worse). This assessment scale was originally developed for psychological purposes but is currently used in other medical fields for assessing the improvement of pain.^[23]^

PGIC will be measured twice in total; at week 3 (visit 8) and week 8 (visit 9).

##### 2.4.2.6 Credibility and expectancy

For the assessment of the expectancy for treatment of the patients, a 9-point Likert scale will be used.

During the first visit at Week 1 (baseline), the subjects will select a score in response to a question “How much do you think the treatment you receive during the clinical trial period will relieve your symptoms?” (1 = almost no expectancy, 5 = moderate, and 9 = highly expectant).

##### 2.4.2.7 Physical examination, ROM

Muscle weakness and loss of sensation will be examined and recorded. The active ROMs for the neck for flexion, extension, lateral flexion, and rotation will also be measured. In addition, the pain and discomfort status during each movement will be measured and recorded.

Physical examination and measurement of ROM will be performed 6 times in total; at the baseline, the last visit of each week during the intervention period (visits 4 and 7), week 3 (visit 8), and week 8 (visit 9).

##### 2.4.2.8 Five-Level EuroQol 5-dimension (EQ-5D-5L)

EQ-5D indirectly estimates the quality weight of a specific health condition through a survey of the health condition through multiple aspects. It is the most widely used tool for assessing the quality of life. EQ-5D-5L is composed of 5 items (mobility, self-care, usual activities, pain, and anxiety/depression) that investigates the current health status. The subjects respond to the questions using the 5-point Likert scale. (1 = I have no problems about, 2 = I have slight problems about, 3 = I have moderate problems about, 4 = I have severe problems about, and 5 = I am unable to about) In this study, the Korean version EQ5D-5L will be used with its validity verified.^[24]^

EQ-5D-5L will be measured 3 times in total; at the baseline, week 3 (visit 8), and week 8 (visit 9).

##### 2.4.2.9 Twelve-Item short-form health survey (SF-12)

The SF-12 is a survey questionnaire that assesses the health-related quality of life. The SF-12 consists of 12 questions in 8 domains that evaluates the physical and mental health. Domains related to physical health include physical functioning, role physical, body pain, and general health, and domains related to mental health include vitality, social functioning, role-emotional, and mental health. The higher the score, the better the quality of life. In this study, the validated Korean version of the SF-12 will be used.^[25]^

SF-12 will be measured 3 times in total; at the baseline, week 3 (visit 8), and week 8 (visit 9).

##### 2.4.2.10 EuroQol visual analogue scale (EQ-VAS)

EQ-VAS is one of the tools that assess the quality of life. The patient can indicate their health condition along a continuous vertical line of 100 mm in length, with the bottom end indicating the worst health condition possible and the top end indicating the best health condition possible.^[26]^

EQ-VAS will be measured 3 times in total; at the baseline, week 3 (visit 8), and week 8 (visit 9).

##### 2.4.2.11 Economic evaluation

For the economic evaluation, medical costs, non-medical costs, and cost of loss of productivity will be measured. For evaluation of cost items, a separately developed structured questionnaire will be used. Medical costs include not only the formal medical expenses incurred when using medical services at medical institutions but also the informal medical expenses incurred such as purchasing health foods and medical devices. The expenses associated with the use of medical services, such as transport expenses, patient time expenses, and caregiving service expenses, will be categorized as non-medical expenses. The cost of loss of productivity refers to the cost of economic loss incurred by the inability to participate in labor due to the disease itself or premature death due to the disease. To calculate the loss of productivity costs, the Work Productivity and Activity Impairment Questionnaire (WPAI)^[27]^ will be used and will be converted to costs for use in the economic evaluation.

Medical costs will be measured every visit and productivity loss costs will be measured 4 times in total; at the last visit of each week during the intervention period (visits 4 and 7), week 3 (visit 8), and week 8 (visit 9).

##### 2.4.2.12 Drug consumption

The type and dose of medication (medication prescribed due to current medical history or rescue medication) taken during the study period will be checked through a questionnaire during each visit. For treatments other than the medication taken such as the injections, the number of the treatments will be recorded.

### 2.5 Timeline of participants

The timeline of the study is shown in Table 1.

### 2.6 Sample size estimation

To estimate the number of study subjects, the results of the unpublished pilot study was used. Based on the pilot study, the effect size of MSAT for patients with neck pain is 0.55. When estimating the number of samples while using the *t* test as the primary analysis, the result showed that 106 subjects in total (53 per group) will be required. However, since the analysis of covariance is the primary analysis in this study, using the correlation coefficient of the mean difference between the baseline value and primary endpoint calculated through the pilot study, 0.24, the corrected number of samples was determined to be 100. Considering a dropout rate of 20% and recruitment from 4 institutions, a total of 128 subjects (64 subjects per group) were calculated as the number of samples required to conduct the clinical trial.

The calculation was performed based on the assumption level of significance at α=0.05 (2-sided test) and 80% of the power of the test with type 2 error (β) at 0.2. G∗Power 3.1.9.7 was used to estimate the sample size.

### 2.7 Recruitment

Subjects will be recruited using online advertising media and promotional posters, inside, and outside the study institutions.

### 2.8 Randomization and allocation concealment

In this study, a balanced block randomization method will be used. Each study institution will be set as strata, and 2, 4, or 6 will be used at random for the block size. Randomization will be performed by the statistical analysis manager using SAS Version 9.4 (SAS institute. Inc., Cary, NC) to create a random table with the same probability of each individual being selected. When a random table is assigned for each institution, it will be divided into a control group and an experimental group according to the randomization code, and 64 subjects will be assigned to each of the groups.

The generated randomization results will be sealed in envelopes, delivered to each institution, and managed in a cabinet with a double lock. Randomization will be conducted only on subjects who have voluntarily provided written consent after a sufficient explanation of the clinical trial. The randomization envelope will be opened in front of the subject, the subject will be assigned to the randomized group, and the envelope will be stored separately in a cabinet with a double lock. Randomization results cannot be viewed in advance and cannot be changed after assignment

### 2.9 Blinding

Due to the nature of the intervention in this study, it is impossible to blind the physicians and patients. However, in this study, the statistical analysis manager and the outcome assessor will be blinded to secure objectivity and reduce the bias as much as possible. Outcome assessment will be performed in a separate space before the treatment is performed by an assessor, who will not participate in the treatment and does not know the patient's group assignment, and has received standardized training for the data collection procedures.

### 2.10 Data collection and management

In this study, an electronic Case Report Form (e-CRF), based on the internet-based clinical research management systems operated by the Korea Disease Control and Prevention Agency, will be used. Before commencing the study, standard operating procedures (SOP) will be developed, and training will be provided for investigators in each of the institutions about the detailed SOP and e-CRF writing guidelines. Double data entry verification will be conducted to ensure the accuracy of the data entered into the e-CRF and the investigators of each institution will be responsible for the accuracy of the entered data. Data entered in the e-CRF will be cleaned, locked, and concealed from all investigators, except for the investigator in charge of data management.

### 2.11 Statistical analysis

This study will be a randomized controlled study that analyzes the pain intensity scale, functional scale, quality of life, and costs data results of patients who meet the selection criteria.

The primary analyses will be the intention-to-treat analysis, which evaluates those who have received treatment at least once, and the per-protocol analysis, which evaluates only those who have successfully completed the clinical study, excluding those who have been dropped out; the 2 analyses will be performed concurrently.

For processing the missing values, multiple imputations using the Markov Chain Monte Carlo method will be used as the primary method, and 20 imputed datasets will be generated. In addition, the last observation carried forward method will be used for the sensitivity analysis.

The sociodemographic characteristics and treatment expectancy of the subjects will be evaluated for each of the groups. Continuous variables will be represented as mean (standard deviation) or median (quartiles), and categorical variables will be represented as a frequency (%).

The effectiveness endpoint of this clinical trial will be the difference in the amount of change between baseline and each time point by the group for the continuous outcomes (NRS, VAS, NDI, NPQ, EQ-5D-5L, and SF-12). Analysis of Covariance will be performed using the baseline values of each variable as covariates and the group as a fixed factor.

For the sensitivity analysis, datasets without the imputed values will be used to analyze the degree of change from the baseline. The mixed-effects repeated measures analysis will be performed for the primary and secondary outcomes at all time points. The time variable will be included as a categorical variable and the interaction terms of group and time will be included at each time point to evaluate the difference in the effectiveness of MSAT.

To compare the total amount of difference between 2 groups for each outcome within the treatment period (2 weeks) and the entire study period (8 weeks), the areas under the curve (AUC) within each period after randomization will be calculated. The difference in AUC between the 2 groups will be compared using the independent student *t* test.

Survival analysis for VAS of neck pain will be performed. At each time point, the patient ratio will be comparatively analyzed for each time point when the neck pain VAS falls below half of the baseline. A Kaplan–Meier survival analysis will be used to estimate the time until less than half of the neck pain recovery occurs after randomization, and the differences between groups will be compared using a log-rank test. Also, the Cox model will be used to estimate the hazard ratio.

All statistical analyses will be performed using the SAS version 9.4 statistical package (SAS Institute, Cary, NC) with the significance level set to *P* < .05. In addition, the analysis will be performed after the end of the study (when all patients follow-ups have been completed) and interim analysis will not be allowed.

In addition, this study aims to investigate the cost-effectiveness between MSAT and acupuncture treatment through an economic evaluation. The primary economic endpoint will be the incremental cost-effectiveness ratios of cost per Quality Adjusted Life Years (QALY) of the MSAT group against the acupuncture treatment group. The analysis period is the entire study period including the intervention period and follow-up period, and if estimation for the subsequent period is required, the costs and effectiveness after the follow-up period will be estimated by extrapolation through a regression model or a secondary analysis with decision modeling analysis.

Medical costs will be calculated based on the number of treatments and the unit cost. For the unit cost, data on health insurance costs, institutional cost data, and patient answers will be used. The quality of life estimation for the QALY calculation, the quality of life derived by EQ-5D-5L will be used as the main endpoint, and EQ-VAS, and SF-6D will be used for the sensitivity analysis. The AUC method will be used for calculation. The units for the costs will be unified in Korean currency (won) as of 2020 and a 5% discount rate will be applied based on the guidelines for the economic evaluation of the Health Insurance Review and Assessment Service and represented in US dollars using the published exchange rate.

Considering the characteristics of non-parametric data, 95% CI of the incremental cost-effectiveness ratios of cost per QALY will be estimated using the bootstrapping technique. In addition, the probability that of MSAT being cost-effective will be examined using the cost-effectiveness acceptability curves graph according to the degree of willingness to pay. All analyses will be made for costs through the healthcare system and social perspectives.

### 2.12 Adverse events

In this study, all adverse events occurring during the clinical trial will be examined and recorded. The adverse event refers to any undesirable and unintended signs (e.g., abnormalities in the laboratory test results), symptoms, or diseases that appear after the treatment in the clinical trial process, and is not necessary that the adverse event has a causal relationship with the treatment. Adverse events will be collected through patient complaints or observations, and the occurrence of adverse events between groups will be examined by occurrence frequency.

The investigator will evaluate the causal relationship between the respective treatment method and the adverse events that occurred on a 6-point scale (1 = definitely related, 2 = probably related, 3 = possibly related, 4 = probably not related, 5 = definitely not related, and 6 = unknown). In addition, the severity will be categorized into 3 levels using Spilker classification method.

1. Mild did not impair the participant's normal activities of daily living (ADLs), caused minimal discomfort and required no additional treatment
2. Moderate: significantly impaired the participant's normal ADLs and may have required treatment but they were resolved after treatment, and
3. Severe: severely impaired the participant's normal ADLs, required intense treatment, and left sequelae)

### 2.13 Data monitoring

Monitoring will be conducted to ensure the safety of the study's subjects and the integrity of the study data. Monitoring will be conducted through a total of 3 monitoring visits after the introductory meeting from the first subject screening time in the order of 2 regular monitoring visits, and 1 end-of-study monitoring visit at the end of the study. The monitoring will be conducted by the monitoring personnel in the host institution. Monitoring will be conducted by collating and comparing the case report forms and supporting documents, and examining the safety matters of the study's subjects.

Monitoring is planned to be conducted a total of 3 monitoring visits, 2 regular monitoring visits from the time of initial patient enrollment, and one end monitoring visit at the time of completion of the trial.

### 2.14 Ethical consideration and dissemination

#### 2.14.1 Ethical approval

Before initiating the study, the study protocol, CRF, informed consent form, and patient recruitment announcement will be submitted to the IRB at each site to obtain the appropriate approval for the study (JASENG 2020–07–014, JASENG 2020-07-015, JASENG 2020-07-016, and JASENG 2020-07-017). All modifications to the protocol, CRF, informed consent form, and others will be subject to the approval of the IRB. All modifications will be updated on the trial registry. All clinical investigators participating in this study will be educated about the Helsinki Declaration and the Korean Good Clinical Practice Guidelines, the study protocol, and the SOP to protect the patients participating in this study.

#### 2.14.2 Patient consent

Before the start of the clinical study, the investigator will provide sufficient information on the overall processes of clinical trial and treatment in a face-to-face meeting with the patient. The Investigator will obtain a signed informed consent from the patient and provide a copy of the consent form to the patient.

#### 2.14.3 Dissemination

The results of this study will be shared with the participants, healthcare professionals, and the public by publishing or through trial registry. Only investigators who were directly or indirectly involved in the study will be listed as authors on study-related publications. The datasets used in the study are available from the corresponding author on reasonable request.

### 2.15 Confidentiality

All personal information data of study subjects participating in this study will be managed under the strict supervision of the IRB and the personal information of the study subjects will be kept strictly confidential. All data collected from the subjects, that provided their consent to participate in this study, will be treated anonymously. In case of sharing this information with other institutions for research purposes, they may be provided in the form of arbitrary codes after excluding personal information.

### 2.16 Ancillary and post-trial care

In the case of direct harm in relation with this study, appropriate medical care may be obtained as determined by the investigator. Compensations for any damage would be proceeded according to the pre-designated insurance policy related to the study. All participants will be provided with an emergency contact number to reach study investigators so that they can receive the necessary support when they have any question or problem.

## 3 Discussion

For spinal disease, the treatment that utilizes passive and active movements of the patient after needle insertion has gained recent widespread usage in China as well as in Korea.^[13,14]^ Liu et al^[28]^ and Lin et al^[29]^ reported greater improvements in pain and ROM in the MSAT group compared to the control group with other types of treatments such as loxoprofen, physical therapy, and conventional acupuncture in RCT for acute low back pain patients. Shin et al^[30]^ reported greater effective improvement of pain and physical functionality in the MSAT treatment group compared to the diclofenac injection group in their study with patients with severe, acute low back pain. Luo et al^[31]^ reported superior outcomes in the MSAT group compared to the conventional acupuncture treatment group in a short-term treatment in their clinical study on cervical spondylosis. In an RCT with 100 WAD patients from traffic accidents conducted by Kim et al,^[15]^ the group that underwent a combination of trapezius MSAT and integrative Korean medicine treatment demonstrated a faster pain reduction and ROM improvements than the group with integrative Korean medicine treatment alone. However, qualitative evidence for the effectiveness and safety of MSAT is still insufficient.

To our knowledge, this study is the first well-designed, multi-centered RCT that evaluates the effectiveness, safety, and cost-effectiveness of MSAT for acute neck pain. Due to the nature of the study design, the physician and patients cannot be blinded, but the outcome assessors and statistical analysts will be blinded to retain objectivity as much as possible.

The results of this study will be useful for clinicians in primary medical institutions, that are frequently visited by patients with acute neck pain, and for policymakers working in healthcare insurance.

## Author contributions

**Conceptualization:** Doori Kim, Yoon Jae Lee, In-Hyuk Ha.

**Investigation:** Yoon Jae Lee, Kyoung Sun Park, Suna Kim, Ji-Yeon Seo, Hyun Woo Cho.

**Methodology:** Doori Kim, Yoon Jae Lee, Kyoung Sun Park.

**Supervision:** In-Hyuk Ha.

**Writing – original draft:** Doori Kim.

**Writing – review & editing:** Yoon Jae Lee.
